# Using conservation genetics to prioritise management options for an endangered songbird

**DOI:** 10.1038/s41437-023-00609-6

**Published:** 2023-04-05

**Authors:** Fernanda Alves, Sam C. Banks, Max Edworthy, Dejan Stojanovic, Naomi E. Langmore, Robert Heinsohn

**Affiliations:** 1 Division of Ecology and Evolution, Research School of Biology, Australian National University, Canberra, ACT Australia; 2 Fenner School of Environment and Society, Australian National University, Canberra, ACT Australia; 3 Research Institute for the Environment and Livelihoods, College of Engineering, IT and the Environment, Charles Darwin University, Darwin, NT Australia

**Keywords:** Conservation biology, Structural variation

## Abstract

Genetic data can be highly informative for answering questions relevant to practical conservation efforts, but remain one of the most neglected aspects of species recovery plans. Framing genetic questions with reference to practical and tractable conservation objectives can help bypass this limitation of the application of genetics in conservation. Using a single-nucleotide polymorphism dataset from reduced-representation sequencing (DArTSeq), we conducted a genetic assessment of remnant populations of the endangered forty-spotted pardalote (*Pardalotus quadragintus*), a songbird endemic to Tasmania, Australia. Our objectives were to inform strategies for the conservation of genetic diversity in the species and estimate effective population sizes and patterns of inter-population movement to identify management units relevant to population conservation and habitat restoration. We show population genetic structure and identify two small populations on mainland Tasmania as ‘satellites’ of larger Bruny Island populations connected by migration. Our data identify management units for conservation objectives relating to genetic diversity and habitat restoration. Although our results do not indicate the immediate need to genetically manage populations, the small effective population sizes we estimated for some populations indicate that they are vulnerable to genetic drift, highlighting the urgent need to implement habitat restoration to increase population size and to conduct genetic monitoring. We discuss how our genetic assessment can be used to inform management interventions for the forty-spotted pardalote and show that by assessing contemporary genetic aspects, valuable information for conservation planning and decision-making can be produced to guide actions that account for genetic diversity and increase chances of recovery in species of conservation concern.

## Introduction

Genetic diversity is critical for maintaining evolutionary potential and resilience, allowing species to adapt to environmental changes (Willi et al. 2006; Markert et al. 2010; Smith et al. 2014). As small and fragmented populations are prone to genetic processes that can increase extinction risk (e.g., inbreeding depression; Carlson et al. 2014; Smith et al. 2014; Frankham et al. 2019), incorporating genetic factors in the management of endangered species can inform actions to avoid loss of genetic variation, increasing the chances of recovery (e.g., Ottewell et al. 2016; Weeks et al. 2017). However, despite its importance, genetic parameters and genetic management remain one of the most neglected aspects in species recovery plans (Laikre et al. 2010; Taylor et al. 2017; Ralls et al. 2018). This gap stems in part from challenges in translating genetic information into management actions (Taylor et al. 2017). Furthermore, conservation practitioners and conservation genetics researchers may sometimes lack a clear understanding of how genetic tools can assist in practical conservation efforts, resulting in missed opportunities for better-informed management decisions.

Genetic data can answer questions relevant to conservation efforts (Manel et al. 2003; Frankham 2010a, b). For example, dispersal and connectivity are difficult to measure directly by studying marked individuals at large spatial scales, but genetic techniques can overcome this challenge (Koenig et al. 1996; Manel et al. 2005; Schwartz et al. 2007). Understanding population connectivity is crucial to define ‘management units’—population units identified within species to help guide management and conservation (Fraser and Bernatchez 2001; Palsbøll et al. 2007; Funk et al. 2012). The two most common units used in conservation are evolutionarily significant units, representing populations that need to be managed separately because of high genetic and ecological distinctiveness (Allendorf et al. 2012), and management units, which are defined as demographically independent populations (Moritz 1994; Funk et al. 2012). However, we argue that there cannot be a single genetic definition of a management unit because their definition should account for the management question (e.g., Taylor and Dizon 1999). Here we define management units based on both genetic diversity (i.e., genetically differentiated) and dispersal patterns (i.e., dispersal or lack of it between populations). These management units can inform the appropriate scale for demographic and genetic monitoring, or identify the likely origins of recruits for population growth following conservation initiatives. In addition to understanding population dynamics, quantifying genetic diversity within and among populations for management is crucial because these parameters play a key role in fitness and population viability (Frankham 2010a, b).

Assessing genetic parameters can help prioritise populations for conserving genetic diversity and inform management interventions. For example, populations with high genetic diversity might be particularly important for conservation (e.g., higher chance to adapt to environmental changes), and those with low diversity or evidence of inbreeding might require genetic management (e.g., augmented gene flow; Frankham et al. 2019). In species for which conservation translocation (i.e., deliberate movement of organisms from one site to another; IUCN/SSC 2013) is advised, a genetic assessment can inform where founders should be sourced from to guarantee genetic representation and for planning reinforcement regimes to increase effective population size and preserve genetic diversity (Weeks et al. 2015). By conducting a genetic assessment, conservation practitioners can implement genetic management if required or establish genetic monitoring and focus on strategies that mitigate threats and factors limiting population growth with the aim of increasing effective population size (e.g., habitat restoration; Ottewell et al. 2016).

**Fig. 1:**
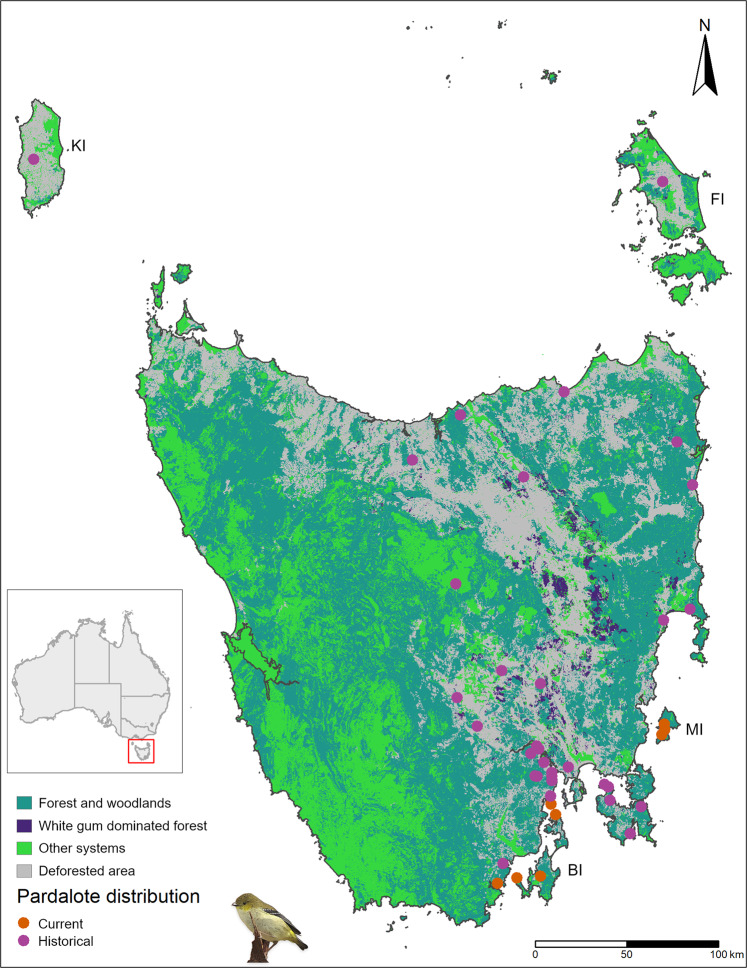
Current (orange dots) and known historical (pink dots) locations of forty-spotted pardalote across Tasmania, showing mapped white gum-dominated forest (pardalote’s preferred food tree) within remaining forest cover according to TASVEG 4.0 (Department of Primary Industries, Parks, Water and Environment, 2020). Offshore islands: KI = King Island, FI = Flinders Island, MI = Maria Island, BI = Bruny Island.

Here, we use genomic data to investigate population parameters and genetic diversity of an endangered songbird to inform conservation management. The forty-spotted pardalote *Pardalotus quadragintus* is a habitat specialist endemic to the island state of Tasmania, Australia (Fig. 1). The species relies on tree cavities for nesting and forages primarily on one tree species, the white gum (*Eucalyptus viminalis*). Forty-spotted pardalotes have been extirpated across most of their former range, mainly due to deforestation and habitat degradation (Brown 1986; Threatened Species Section 2006), and are now largely confined to two offshore islands (Bruny and Maria Islands) and two small patches of forest on coastal mainland Tasmania close to the Bruny Is. population (hereafter southern populations; Fig. 1). Threats limiting remaining populations include ongoing habitat degradation, low nest site availability, competitors, and parasitism by the larvae of an ectoparasitic fly (*Passeromyia longicornis*) that causes severe nestling mortality (Threatened Species Section 2006; Edworthy 2016a, b; Edworthy et al. 2019). Although the species has a recovery plan, most of the actions achieved so far are related to the protection of remaining habitat, revegetation and public awareness (Threatened Species Section 2006). However, given the current limiting and threatening processes present in the remaining populations, understanding population parameters (e.g., genetic diversity, connectivity) is paramount to inform management interventions. For example, reintroduction to mainland Tasmania has been proposed for the species (Webb et al. 2019), but without information on genetic parameters, we cannot assess whether harvesting individuals for such a risky management option would be harmful to the remaining populations (e.g., Morrison et al. 2020). Furthermore, an outstanding question is whether the small mainland populations in southern Tasmania are connected by migration to the larger Bruny Is. population or are isolated remnants of the species’ historical distribution. Small populations are particularly prone to be negatively impacted by harvesting due to their often-small effective population size (Allendorf et al. 2012). Thus, a genetic assessment can provide valuable information to inform management options for species that have suffered severe range contraction, such as the forty-spotted pardalote.

**Fig. 2:**
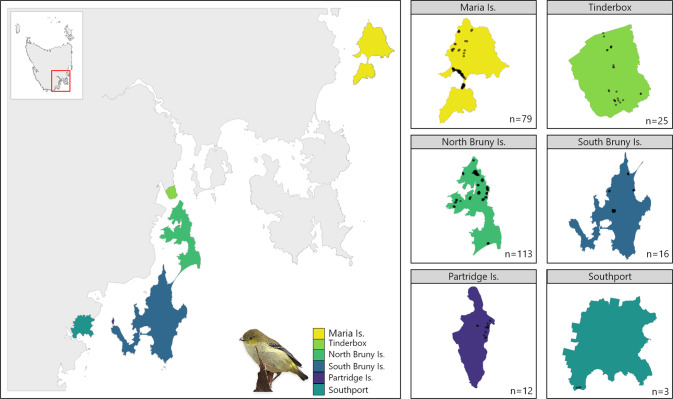
Spatial distribution of the six sampled forty-spotted pardalote populations in eastern Tasmania. Small panels on the right show each location with sample size and black dots represent sampled individuals.

We collected DNA samples across known remaining populations (Fig. 2) and used genotyping-by-sequencing to undertake the first comprehensive genetic assessment of the species. Because of the fragmented nature of the remaining populations, we expected them to show genetic structure, and based on this prediction, we aimed to address the following questions: How is genetic diversity distributed within and among populations? Do some populations have lower genetic diversity than others? Which populations are most important for maintaining genetic diversity in the species? How many management units are necessary to conserve genetic diversity and facilitate recovery? What is the effective population size (*N̂*_*e*_) within management units? Are the smaller populations adjacent to Bruny Island discrete populations, and possibly at risk of extinction, or are they connected by migration? Answering the latter question will clarify the extent to which the species can colonise new areas if habitat and threatening processes are better managed. By answering these questions, we can make recommendations on management strategies that account for genetic diversity and the harmful genetic effects common in small populations.

## Methods

### Sample collection

We collected samples from three offshore Islands (Bruny Is., Maria Is. and Partridge Is., a small island just offshore from the southwestern coast of Bruny Is.) and from two sites on mainland Tasmania (Tinderbox Peninsula and Southport, where a small population was rediscovered in 2015; Fig. 2). South Bruny Is. and North Bruny Is. are isolated by an isthmus (~7 km) of inhospitable habitat, so we treated them as separate population units. Maria Is. is also divided by an isthmus (700 m), but it is smaller and supports pardalote habitat (i.e., forest where white gums are present), so we did not consider populations on either side of the isthmus to be geographically isolated. These sites comprise all known remaining populations of the species (Fig. 2), with the possible exception of Flinders Island in the Bass Strait where pardalotes were last recorded in 2012 at very low numbers (Webb et al. 2019) and may be functionally extinct (Fig. 1).

We sampled nestlings and adult birds between 2012 and 2019. Nestling samples were collected during nest monitoring for other studies (e.g., Edworthy et al. 2019; Alves et al. 2020) and adult birds were captured using mist-nets. To ensure the same individual was not resampled, we banded all birds using bands supplied by the Australian Bird and Bat Banding Scheme. We collected blood using brachial venepuncture in accordance with guidelines approved by Australian National University Animal Ethics Permits 2012/34 and A2017/38 and Tasmanian Government Scientific Permits TFA13956, TFA14295, and TFA18255. Blood was stored either in 70% ethanol or on FTA paper (WhatmanTM). We collected 316 samples across the populations but for this study, when samples were collected from a brood in a nest, we only kept one sample from each nest to avoid the impact of relatedness (i.e., siblings) on the parameters we sought to estimate (Wang 2018). Therefore our dataset comprises 248 samples (Maria Is. *n* = 79, Tinderbox Peninsula *n* = 25, North Bruny Is. *n* = 113, South Bruny Is. *n* = 16, Partridge Is. *n* = 12; Southport *n* = 3; Fig. 2).

### DNA extraction, SNP genotyping and exploratory analysis

DNA extraction and single-nucleotide polymorphism (SNP) genotyping were performed by Diversity Arrays Technology Pty. Ltd. (DArT; Canberra, Australia; https://www.diversityarrays.com/), using DartSeq™ protocols, which are independent of reference genome sequence availability (Jaccoud et al. 2001; Kilian et al. 2012). DartSeq is a genome complexity reduction technology that digests genomic DNA using pairs of restriction enzymes (cutters). We first plotted histograms of basic SNP metrics including call rate (i.e., the proportion of non-missing data per SNP), repeatability based on technical replication of 30% of samples and sequencing depth, and used scatterplots to visually assess whether these locus-level metrics were associated with expected and observed heterozygosity and F_IS_ within the larger populations sampled (Supplementary Figs. S1–S3). We then filtered the data and calculated genetic diversity metrics using the ‘dartR’ package v2.0.4 in R (Gruber et al. 2018; R Core Team 2021). DArT’s sequence processing and SNP-calling produced 33,831 SNPs loci, of which 12,699 SNPs were retained after filtering (no individual samples were dropped during filtering). We filtered SNPs by repeatability (95% repeatability; 27,630 SNPs retained; Supplementary Fig. S4), removing monomorphic loci (27,319 SNPs retained), minimising missing data (per-locus call rate >0.95; 15,670 SNPs retained; Supplementary Fig. S5), dropping secondary SNPs in the same sequence (retaining the SNP with highest polymorphic information content; 14,940 SNPs retained), and on individuals by the amount of missing data (call rate >0.90; 14,940 SNPs retained; Supplementary Fig. S6). We also filtered on pairwise Hamming distance (i.e., loci with trimmed sequence tags that are too similar; 12,699 SNPs retained; Supplementary Fig. S7). Using the filtered dataset, we calculated overall genetic diversity statistics (i.e., *H*_*o*_, *H*_*e*_, *F*_*ST*_, *F*_*IS*_) and pairwise *F*_*ST*_ with 100 bootstraps (Gruber et al. 2018). For some analyses, further filtering was conducted (see below).

### Population genetic structure

As an exploratory step, we conducted a principal coordinate analysis (PCoA) using package ‘dartR’ (Gruber et al. 2018) to visualise the underlying population structure. Then, we further investigated population structure using two methods. We first conducted a discriminant analysis of principal components (DAPC) using package ‘adegenet’ v.2.1.8 (Jombart 2008; Jombart and Ahmed 2011). DAPC identifies and describes clusters of genetically related individuals from large datasets by summarising the genetic differentiation between groups while overlooking within-group variation, therefore achieving the best discrimination of individuals into pre-defined groups (Jombart et al. 2010). We first assumed 1–8 clusters (*K*) and used Bayesian Information Criterion to assess the best-supported model and identify the optimal value of *K* to evaluate population groupings (Jombart et al. 2010).

We also analysed population structure using the Bayesian clustering method in the STRUCTURE program (Pritchard et al. 2000) implemented via the R package strataG v2.5.01 (Archer et al. 2017). For this analysis, we further filtered the dataset on loci based on linkage disequilibrium (LD) using the function *gl.filter.ld* in dartR (12,269 loci were retained after filtering; Gruber et al. 2018). Using the *structureRun* function (Archer et al. 2017), we ran a model with the following parameters: correlated allele frequency among populations, a burn-in period of 100,000 and 100,000 MCMC iterations, and used the admixture model. STRUCTURE can yield erroneous inferences when samples from different populations are uneven (e.g., Kalinowski 2011; Puechmaille 2016), but changing some parameters can overcome this issue (Wang 2017). Since our sample was unbalanced, we followed Wang (2017) and set the parameter alpha to 1.0/K (1.0/8 = 0.125; we searched for up to eight ‘populations’), and used the alternative ancestry prior (uniprioralpha = 0). We ran analyses for *K* = 1–8, each replicated 20 times and used diagnostic plots of number of groups (*K*) and first- and second-order changes in LnP(*K*) as described in Evanno et al. (2005) to compare support for different numbers of clusters. We then used function CLUMPP in strataG (Archer et al. 2017) to average the results and minimise variance across iterations (Jakobsson and Rosenberg 2007).

### Identification of immigrants

To identify the individual origin and investigate possible source-sink dynamics, we conducted an individual genetic assignment analysis using the R package ‘rubias’ v.0.3.0 (Moran and Anderson 2018). We used the function *self_assign*, which assigns individuals to the population of origin using a *leave-one-out* procedure. Working through each individual in the dataset, the method removes an individual and uses the remaining individuals as a reference panel to calculate the likelihood of that genotype arising in every possible source population. It reports likelihoods for ‘reporting units’, which can be the same as the candidate source populations or a collection of local populations combined (Moran and Anderson 2018). We first ran an analysis keeping all our source populations (Maria Is., Tinderbox, North Bruny Is., South Bruny Is., Partridge Is.) as reporting units, except for Southport where there were too few samples. North Bruny and South Bruny were separated based on results from STRUCTURE and DAPC and we kept Tinderbox and Partridge Is. separate from North Bruny and South Bruny, respectively, because we wished to quantify dispersal among these locations. We also conducted an analysis in which we combined individuals from Partridge Is. and South Bruny Is. into one reporting unit (i.e., South Bruny) given their proximity and results from STRUCTURE and DAPC.

### Local effective population size (*N̂*_*e*_)

We estimated local effective population size using the single-sample bias-corrected LD method (Waples 2006; Waples and Do 2010) using the software NEESTIMATOR v2.1 (Do et al. 2014) via the function *gl.LDNe* in dartR (Gruber et al. 2018). For this analysis, we filtered the SNPs by 100% repeatability (4393 SNPs retained). To account for the population structure we found (see results; Luikart et al. 2010), we combined individuals from South Bruny Is., Partridge Is. and Southport into one group (i.e., South Bruny), Tinderbox and North Bruny into another group (i.e., North Bruny) and kept Maria Is. separated. Because Tinderbox is a geographically distinct population with different threats and management issues, we re-run the analysis to estimate *N̂*_*e*_ for North Bruny and Tinderbox separately as well. Even though the LD method seems robust to certain levels of migration (Waples 2010), for Tinderbox we removed six individuals that migrated from Bruny Is. identified in the assignment analysis (see results) as they can cause upward bias in local effective population size estimates (Luikart et al. 2010). We conducted analyses under the assumptions of monogamy and random mating. We screened out rare alleles (which can create upward bias in LD estimates) with frequencies below two critical values (*P*_crit_ = 0.02 and 0.05; Turner et al. 2001; Waples and Do 2010), and calculated confidence intervals using the jackknife method (Waples and Do 2008; Do et al. 2014).

### Genetic conservation prioritisation

We conducted two analyses to inform management strategies by quantifying the contribution of each population to the genetic diversity of the sampled set of populations and identifying the combinations of populations that best represent that diversity, broadly following the approach of von Takach et al. (2021). We first followed Petit et al. (1998) and assessed the contribution of each population to the total allelic richness represented in the 12,699 SNP dataset (across all populations sampled). This method uses rarefaction, which allows evaluation of the expected number of different alleles among equal-sized samples drawn from several different populations (Petit et al. 1998). The sample size for each combination of population and locus is set equal to the smallest number of alleles seen in a sample across all combinations of population and locus (Petit et al. 1998). We focussed on allelic richness because this metric measures the number of alleles per locus and is more dependent on effective population size than average heterozygosity (Leberg 1992). Furthermore, allelic richness is considered a good indicator of evolutionary potential (Caballero and García-Dorado 2013; Greenbaum et al. 2014; Forester et al. 2022).

Based on our population structure analysis (see results), we first combined Partridge Is. and South Bruny Is. into one population (i.e., South Bruny) and removed individuals from Southport due to the small sample size (*n* = 3). For this analysis, we also kept Tinderbox as a separate population as we wanted to assess the effect of losing Tinderbox. We used the function *allel.rich* in *PopGenReport* package v.3.0.7 (Adamack and Gruber 2014) and estimated mean allelic richness per locus for a standardised sample of 25 biallelic individuals (equivalent to the smallest sample size at Tinderbox) from each population. Then, we iteratively removed one population at a time from the dataset to estimate the proportional loss of allelic richness that would result if any one of these populations went extinct, using the formula AR(*t*) – AR(–*i*) / (AR(*t*) – 1). Where AR(*t*) is total allelic richness and AR(–*i*) is allelic richness over all populations excluding the one in question (Petit et al. 1998).

We also used MARXAN software, which uses stochastic optimisation routines to solve a range of conservation prioritisation problems (Ball et al. 2009). This method identifies networks of extant populations that best represent the total genetic diversity in the species, as estimated by the sampled populations across the 12,699 SNP loci. For this exercise, we did not have a specific cost for the conservation options, so we allocated an equal unit cost for the conservation of each population. Using the function *marxan_problem* in *prioritizr* package v 5.0.2 (Hanson et al. 2020) and the *lpsymphony* solver (Kim 2022), we identified the optimal network of populations to maximise allelic richness in the species. We identified optimal solutions for scenarios of 1, 2, 3 or 4 ‘protected’ populations, meaning that in scenario 1, for example, if we were to conserve only one population, which one would best represent genetic diversity across populations. We also calculated the proportion of total alleles represented in each solution. Although we recognise that all populations are important for conservation, these analyses provide metrics of the unique contribution of each population to genomic diversity in the species to inform strategies for conservation.

## Results

### Genetic diversity

Overall observed (*H*_*o*_) and mean expected (*H*_*e*_) heterozygosity were estimated at 0.21 and 0.23, respectively. Overall, *F*_*IS*_ (population-level inbreeding coefficient) was estimated at 0.10 and *F*_*ST*_ (fixation index) at 0.9. *H*_*o*_ values were similar across populations, except for the more isolated population on Maria Is., which had lower *H*_*o*_ and *H*_*e*_ (observed and expected heterozygosity, respectively) (Table 1). Pairwise *F*_*ST*_ values indicate that Maria Is. is the most distinct population, while the southern populations are similar, showing connectivity among these populations (Table 2).

**Table 1:** Estimated observed heterozygosity (*H*_*o*_), and expected heterozygosity (*H*_*e*_) with standard deviations (*H*_*o* SD_; *H*_*e* SD_), and inbreeding coefficient (*F*_*IS*_) with confidence intervals (CI) for each geographic area sampled over a total of 12,699 variable SNPs across sites.

Location	Sample size	Number of polymorphic loci	*H*_*o*_	*H*_*o* SD_	*H*_*e*_	*H*_*e* SD_	*F*_*IS*_ (CI)
Maria Is.	79	9164	0.18	0.18	0.19	0.19	0.08 (0.07–0.08)
North Bruny Is.	113	12,099	0.22	0.16	0.25	0.16	0.10 (0.09–0.10)
Partridge Is.	12	9235	0.21	0.20	0.22	0.18	0.09 (0.08–0.10)
South Bruny Is.	16	9975	0.21	0.18	0.23	0.18	0.11 (0.10–0.12)
Southport	3	6432	0.21	0.27	0.19	0.20	0.09 (0.10–0.13)
Tinderbox	25	10,800	0.22	0.17	0.24	0.18	0.11 (0.10–0.11)

**Table 2:** Pairwise estimates of genetic differentiation (*F*_*ST*_), between forty-spotted pardalote populations.

	Maria Is.	Tinderbox	North Bruny Is.	South Bruny Is.	Partridge Is.
Maria Is.					
Tinderbox	0.23				
North Bruny Is.	0.19	0.03			
South Bruny Is.	0.23	0.06	0.05		
Partridge Is.	0.24	0.07	0.06	0.02	
Southport	0.25	0.06	0.04	0.01	0.01

All *P* values are <0.01.

### Population genetic structure

**Fig. 3:**
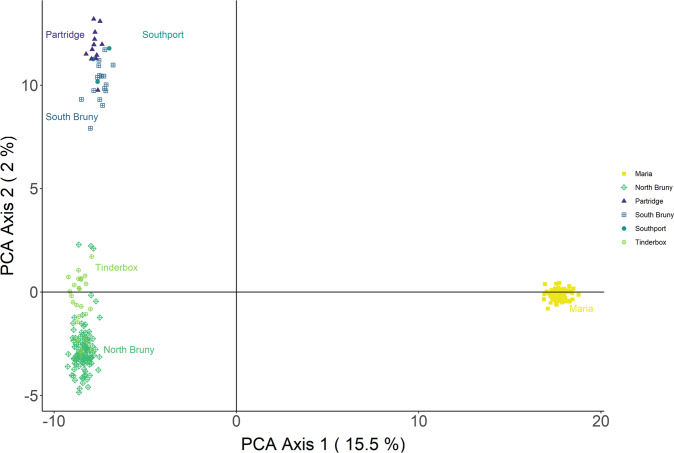
Principal coordinates analysis of individual forty-spotted pardalote SNP genotypes. Individuals are coloured by sampling location.

**Fig. 4:**
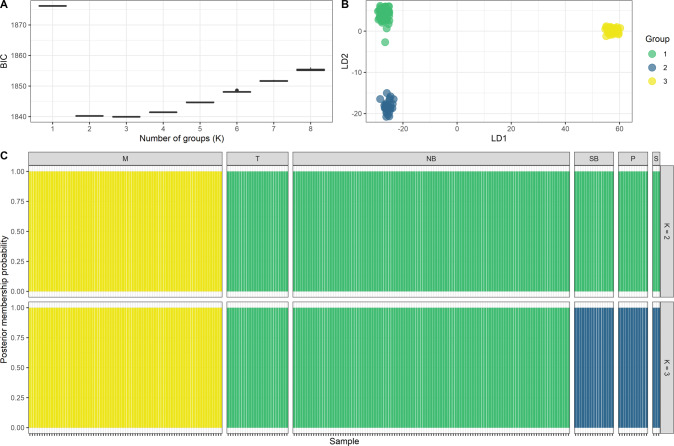
Discriminant analysis of principle components (DAPC). **A** Values of BIC versus number of clusters (K); **B** scatterplot based on the discriminant functions showing four clusters (*K* = 4); **C** bar plots of the posterior probabilities of a group assignment for each sample across *K* explored (each bar represents an individual). Header on plot **C**: M = Maria Is., NB = North Bruny Is., *P* = Partridge Is., SB = South Bruny Is., S = Southport, T = Tinderbox Peninsula.

The first two PCoA axes explained 17.5% of the variance in the dataset and grouped individuals into three distinct clusters (Fig. 3). The first PCoA axis separated the more isolated Maria Is. population from the remaining sampled regions, while the second PCoA axis showed two clusters. Individuals from North Bruny Is. and Tinderbox Peninsula clustered together (mainland site separated by 1.4 km of water), while individuals from South Bruny Is., Partridge Is. and Southport formed another cluster (Partridge is isolated by 0.4 km of water west of South Bruny, and Southport is a mainland site 4.4 km of water west of South Bruny). Similarly, the DAPC showed evidence of population structure with three clusters (*K* = 3), but also showed some support for a *K* = 2 model (Fig. 4).

**Fig. 5:**
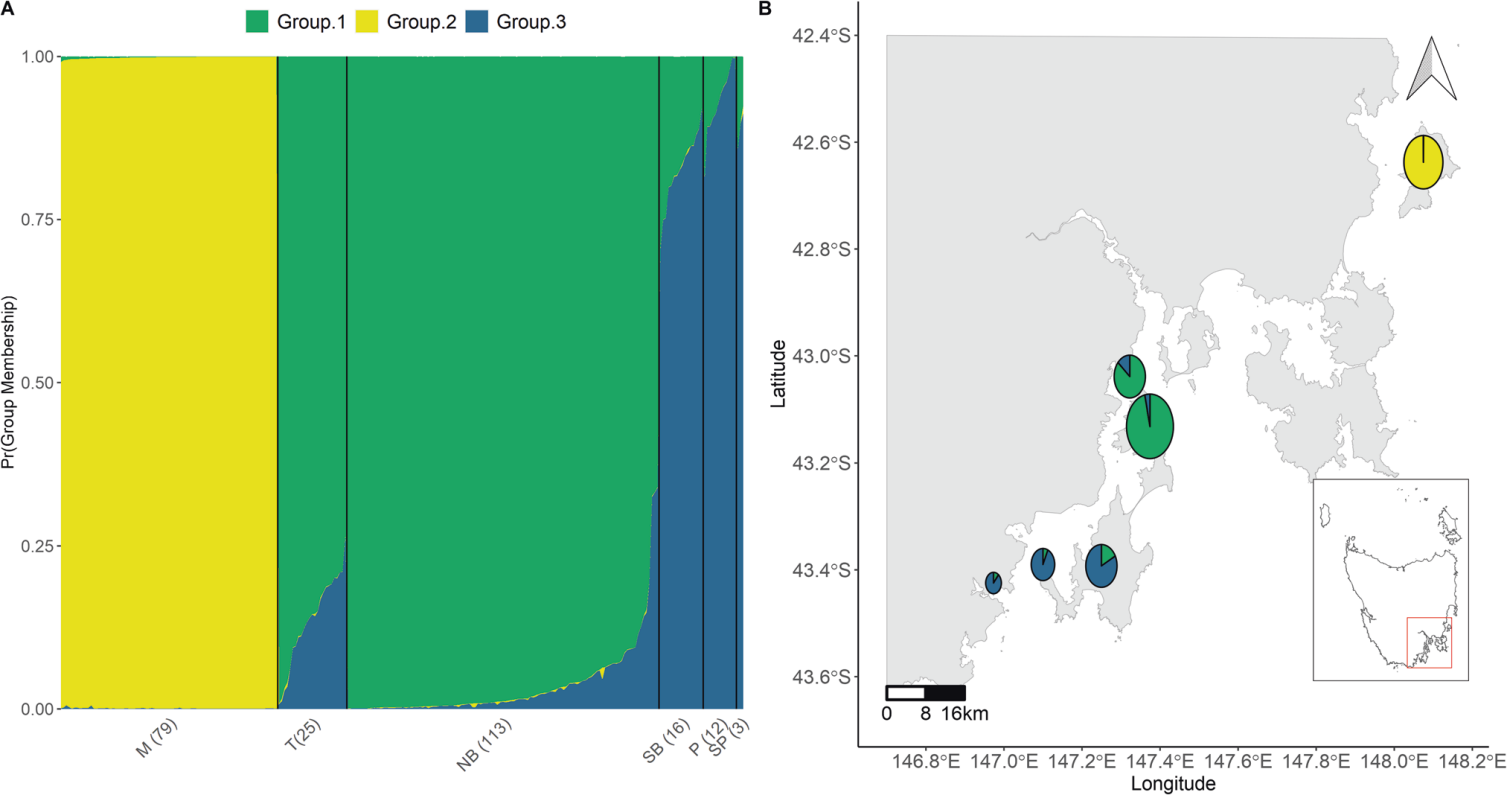
Results of STRUCTURE analysis with correlated allele frequency. **A** Distribution of the probability of group membership for each population. Numbers in brackets represent sample size and plot labels are coded as: M = Maria Is., NB = North Bruny Is., *P* = Partridge Is., SB = South Bruny Is., S = Southport, T = Tinderbox. **B** Each pie chart represents a sampled population (pie chart size reflects sample size shown in plot **A**), and colours the three clusters identified in the STRUCTURE analysis. An interactive plot with individual pie charts and membership probability can be found in Supplementary Fig. S8.

Results from STRUCTURE showed the same pattern of population structure as those suggested by the PCoA and DAPC. STRUCTURE revealed *K* = 2 as the most likely number of genetic clusters (Supplementary Fig. S8 and Fig. 5), with no evidence for contemporary migration between two distinct clusters corresponding to Maria Island and the Bruny Island, Southport and Tinderbox region. However, we have based most of our interpretation on the *K* = 3 model as this was also well-supported (and consistent with DAPC) and suggested some additional genetic population structure occurring between clusters comprising North Bruny and Tinderbox, and South Bruny, Partridge Is. and Southport. Clearly, the ‘major’ structure occurs among Maria Island and the southern populations, but there is no panmixia in the southern region and the information on population structure here, especially the genetic affinities of the smaller Tinderbox, Partridge Is. and Southport individuals to North Bruny and South Bruny, respectively, has relevance for management.

### Identification of immigrants

**Fig. 6:**
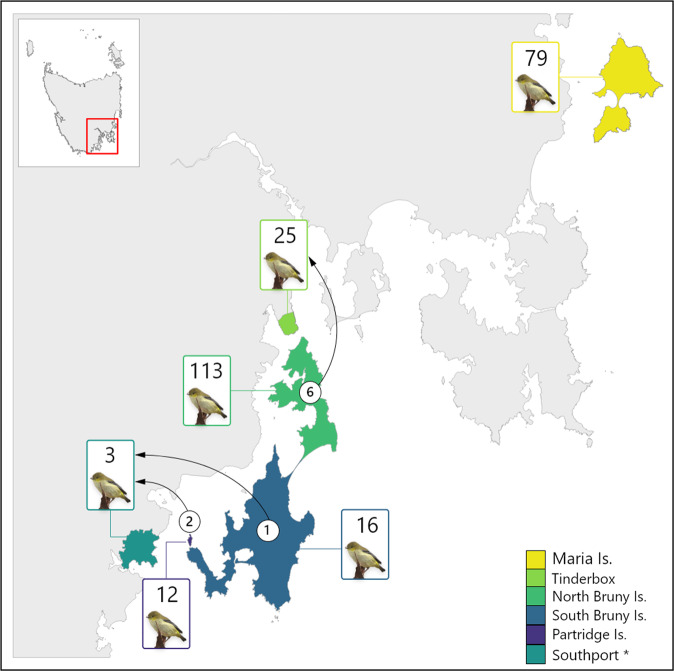
Results of the individual assignment analysis. Except for Southport*, all collection sites were used as reporting units (coloured areas). Boxes show sample size in each reporting unit and arrows indicate dispersal direction with the number of assigned dispersers in the circles.

In the first run of the analysis, in which we kept our geographically sampled populations as reporting units, most individuals were assigned to their reporting unit of origin. Six individuals from Tinderbox were assigned to North Bruny, corresponding to a 24% immigration rate into Tinderbox. All three individuals from Southport were assigned to either Partridge Is. (*n* = 2) or South Bruny Is. (*n* = 1; Fig. 6), but note that we did not include Southport itself as a candidate source population, as only three individuals were found at that site. In the analysis where Partridge Is. and South Bruny Is. were merged into one reporting unit, all individuals from Southport were assigned to the merged unit. All individuals were assigned to the reporting units with probabilities near 1 and therefore were all accepted with high confidence (Supplementary Table S1).

### Local effective population size (*N̂*_*e*_)

Estimates of effective population size (*N̂*_*e*_) were similar across the two critical values used to screen out rare alleles (0.05 and 0.02; Table 3). Maria Is. has the largest effective population size (random mating = 323.4; monogamy = 641.9), followed by the South Bruny Is., Partridge Is. and Southport cluster (random mating = 191.2; monogamy = 381.6), and North Bruny Is. and Tinderbox cluster (random mating = 176.7; monogamy = 349.6). When considered separately, North Bruny Is. *N̂*_*e*_ was estimated at 188.4 for random mating and 348.6 for monogamy, and Tinderbox 33.5 for random mating and 68.4 for monogamy (Table 3).

**Table 3:** Local estimates of effective population size (*N̂*_*e*_) with 95% confidence intervals in brackets for forty-spotted pardalote populations using the linkage disequilibrium method.

	Random mating		Monogamy	
	*P*_Crit_ = 0.05	*P*_Crit_ = 0.02	*P*_Crit_ = 0.05	*P*_Crit_ = 0.02
Maria Is. (*n* = 79)	323.4 (263.2–829.5)	337.9 (210.4–561.1)	641.9 (445.9–1392.1)	685.8 (550.7–2.777.2)
North Bruny Is. + Tinderbox (combined *n* = 138)	176.7 (144–238.3)	187.7 (122–171.3)	349.6 (251–384.3)	370.7 (332.6–547.4)
South Bruny Is. + Partridge + Southport (combined *n* = 31)	191.2 (78–241.3)	205.9 (67.8–147.8)	381.6 (170.4–638.8)	426.7 (397.9–∞)
North Bruny Is. (*n* = 113)	188.4 (132–201.4)	188.1 (153.2–253)	348.6 (306.9–538.2)	380.8 (252.4–371.4)
Tinderbox (*n* = 19)	33.5 (16.8–50.9)	42.9 (24.2–89.4)	68.4 (40.6–164.9)	88.6 (53.1–217.2)

Estimates are presented for both mating systems and for the two critical values used to screen out rare alleles.

### Genetic conservation prioritisation

North Bruny Is. has the highest mean allelic richness, followed by Tinderbox Peninsula, South Bruny Is. and Maria Is. (Table 4). However, in terms of contribution to total allelic richness across the species, the populations that contributed the most were North Bruny and Maria Is. The MARXAN results were similar and identified North Bruny as the single population that contains the highest proportion of the total genetic diversity in the species, and this was the priority population in a scenario where only a single population could be protected. Protecting North Bruny alone represented 97% of the alleles detected in the SNP panel we analysed. The second scenario identified Maria Is. as the next priority and South Bruny as the third priority (Table 4).

**Table 4:** Mean allelic richness with standard deviation for each population of forty-spotted pardalotes and allelic richness contribution following (Petit et al. 1998).

Population	Mean allelic richness	Allelic richness contribution	Solution 1	Solution 2	Solution 3	Solution 4
Maria Is.	1.63 ± 0.44	0.048	0	1	1	1
North Bruny Is.	1.89 ± 0.26	0.051	1	1	1	1
South Bruny Is.	1.81 ± 0.37	0.010	0	0	1	1
Tinderbox	1.83 ± 0.35	0.003	0	0	0	1
Proportion of alleles			0.97	0.99	0.99	1

MARXAN results, showing population identified as a priority in each scenario (solutions 1–4 represent alternate management strategies in which 1, 2, 3, or 4 populations can be protected with available resources), with the proportion of alleles represented in each solution.

## Discussion

In this study, we used genetic data to investigate population parameters relevant to the management of the endangered forty-spotted pardalote. We aimed to address the following questions: How is genetic diversity distributed within and among populations? Do some populations have lower genetic diversity than others? Which populations are most important for maintaining genetic diversity in the species? How many management units are necessary to conserve genetic diversity and facilitate recovery? What is the effective population size (*N̂*_*e*_) within management units? Are the smaller populations adjacent to Bruny Island discrete populations, and possibly at risk of extinction, or are they connected by migration?

Our results revealed an unknown population genetic structure corresponding to geographical barriers and fragmentation. The major structure among the sampled populations of the species occurred between Maria Island and the southern (Bruny Is., Partridge Is., Tinderbox, Southport) populations but there was also evidence of north-south structure within the populations on Bruny Is. and the associated ‘satellite’ populations. We also demonstrate connectivity among the southern populations via migration events and evidence of small effective population size (*N̂*_*e*_ ≤ 100; Frankham et al. 2014), showing that some populations might be vulnerable to genetic stochasticity (Frankham et al. 2010). Our prioritisation analyses showed North Bruny Is. and Maria Is. best representing the remaining genetic diversity in forty-spotted pardalotes. Below, we discuss our results in detail and show that this study system corroborates the importance of integrating genetics into conservation planning for threatened species (Ralls et al. 2018; Hohenlohe et al. 2021).

### Genetic diversity

Our panel of 12,699 SNPs shows similar genetic diversity and low genetic differentiation across Bruny Is. and adjacent populations (*H*_*o*_ range: 0.21–022; *F*_*ST*_ range: 0.01–0.07). The more isolated Maria Is. (74 km NE of the closest mainland population) had slightly lower genetic diversity (*H*_*o*_ = 0.18) and higher differentiation from the other populations (*F*_*ST*_ range: 0.19–0.25) with moderate positive *F*_*IS*_ (*F*_*IS*_ = 0.08). Higher genetic differentiation on Maria Is. reflects the lack of gene flow from other remaining populations. Similar patterns have been described for other passerine species, where higher divergence as a result of limited dispersal due to fragmentation and unsuitable habitat was reported even at smaller spatial scales than seen in our system (e.g., Callens et al. 2011; De Camargo et al. 2015). Unlike some endangered species with fragmented populations and highly structured genetic diversity that require genetic restoration (e.g., Reynolds et al. 1999; Westemeier et al. 1998; Mitrovski et al. 2007; Weeks et al. 2017; von Takach et al. 2021), genetic diversity of forty-spotted pardalotes is represented in most remaining populations, with no immediate need for genetic management. However, we do recommend genetic monitoring for forty-spotted pardalotes, with Maria Island being the priority in the short term due to lower diversity and higher *F*_*IS*_ than the other populations, as genetic data can provide valuable information for management interventions (Schwartz et al. 2007; Carroll et al. 2018).

### Population structure and management units

We identified management units from genetic data in the context of two questions. The first relates to local recruitment dynamics and whether a number of small mainland Tasmanian populations should be considered demographically discrete units for management or whether they are dependent on other areas for recruitment. The second question relates to how best conserve genetic diversity in the species and we addressed this by identifying discrete populations that can be considered discrete units in terms of their contribution to total genetic diversity in the species and for planning potential genetic management within and among those units. The foundational information for both of these management questions was the analysis of the genetic structure and the conservation prioritisation analysis.

We considered a model of three genetic clusters most relevant to conservation planning because it provided information on the migration sources of the small mainland populations over and above that provided by the simpler *K* = 2 model. This model showed genetic structure even within Bruny Is., where two clusters (north and south Bruny) connected to their nearby mainland sites were detected. Migrants from Bruny Is. detected on mainland sites further confirmed connectivity between southern populations and demonstrated that the Tinderbox population receives migrants from North Bruny and the Southport population is dependent on migrants from South Bruny. This stronger connectivity between Bruny Is. and adjacent mainland populations rather than between the north and south portion of the island may be explained by unsuitable habitat and fragmentation of suitable habitat. Forty-spotted pardalote habitat (i.e., forest where white gums are present) on North Bruny Is. is highly fragmented, and the isthmus connecting north to south is long and covered by heath (~7 km of unsuitable habitat). While North and South Bruny Is. populations are not completely distinct (i.e., there is some level of connectivity between north and south), the genetic structure within the island is likely to be driven by unsuitable habitat and potentially compounded by fragmentation of suitable habitat to both the north and south of the isthmus (e.g., Peery et al. 2010; Adams and Burg 2015; Schlaepfer et al. 2018), making dispersal to adjacent mainland sites easier. Although Maria Is. also has an isthmus, it is much smaller (700 m) and mostly covered by forest (including white gums), allowing dispersal between north and south, which is evident in the single genetic cluster we detected on the island.

Both analyses used in our conservation prioritisation approach identified North Bruny and Maria Is. as most important for representing the genetic diversity in forty-spotted pardalotes, with North Bruny alone contributing 97% to total allelic richness. When North Bruny Is. and Maria Is. are combined total richness rises to 99%. Although we do not have data to suggest these populations fulfil the requirements of evolutionarily significant units, they are important distinctive units, and therefore we consider Maria Is. and the southern populations as different units (i.e., 2 management units) for the purposes of conserving genetic diversity in the species as they reflect population units with no migrant interchange. While we recognise Maria Is. and the Southern populations as separate units for conserving genetic diversity, for certain management interventions (see the section on considerations for translocations below), it might be beneficial to mix individuals from these populations. For managing populations and facilitating species recovery, we use the traditional definition of management units (i.e., considering demographic processes) and consider three units based on the structure and migration patterns we found in the southern populations. In this scenario, Maria Is. is a unit on its own, North Bruny Is. and Tinderbox form a second unit, and South Bruny Is., Partridge Is. and Southport form a third unit. The connectivity among the southern populations indicates that forty-spotted pardalotes have some capacity for cross-water colonisation of suitable habitat, which is useful from a restoration perspective. If future genetic monitoring identifies the need for genetic management of forty-spotted pardalotes, translocations between the North Bruny/South Bruny Is. and Maria units would result in the greatest increase in genetic diversity, although at this stage, the relative gain in genetic diversity from such actions is low; therefore, we argue that investing in actions that mitigate current threatening process that impede population growth should be prioritised.

### Local effective population size

Mating system has a major influence on effective population size (Frankham et al. 2010), and although we estimate effective population size for both random mating and monogamy, because pardalotes are socially monogamous, we concentrate on interpreting the results for monogamy. However, it is likely that their true effective population size is somewhere between the two mating systems, given that genetic monogamy (i.e., lifetime pair bond) is rare in passerines (Hasselquist and Sherman 2001).

Effective population sizes were small for the North Bruny Is. cluster (i.e., North Bruny and Tinderbox = 349.6) and for South Bruny Is. cluster (South Bruny, Partridge and Southport = 381.6) in comparison to Maria Is. (*N̂*_*e*_ = 641.9). These results may seem surprising, given that genetic diversity in the southern populations was higher than Maria Is., and populations with smaller effective population sizes are expected to have decreased genetic diversity due to the effects of inbreeding and drift (Frankham 2005). However, the pattern we found here may be explained by a combination of different habitat quality between the southern populations and Maria Is., and the effect of population structure on LD. Recent land-clearing and fragmentation on Bruny Is. has potentially affected population connectivity leading to the structure we found in the southern populations. Given that population structure can affect patterns of LD (e.g., Maccaferri et al. 2005), which is the method we used to estimate *N̂*_*e*_, any recent changes in population size would likely be observed in LD patterns before changes in heterozygosity. The fragmented landscape in which the southern populations occur in combination with other known threatening processes, such as low nesting site availability and reduced breeding success (Edworthy et al. 2019; Alves et al. 2020) likely contributed to the smaller contemporary local population size estimated for these populations. Although Maria Is. is isolated and has lower genetic diversity, the island is a national park dominated by contiguous, mature native forest. This better habitat quality probably allows increased reproductive success and the maintenance of higher effective population size in local forty-spotted pardalotes (e.g., Reed 2004).

Even if the mating system of pardalotes is genetically monogamous (for which local *N̂*_*e*_ values were higher), effective population sizes for all populations are below the threshold required to maintain sufficient evolutionary potential in the long term (*N̂*_*e*_ ≥ 1000) according to Frankham et al. (2014). Furthermore, if their mating system is non-monogamous, the estimate of *N̂*_*e*_ for Tinderbox when considered separated from North Bruny (*N̂*_*e*_ = 33.5) is already below the size needed to minimise short-term problems (*N̂*_*e*_ ≥ 100, Frankham et al. 2014), indicating that some populations are vulnerable to genetic stochasticity (Frankham et al. 2010), and showing the necessity to implement actions that increase populations size. Nonetheless, some studies have shown low *N̂*_*e*_ in a range of island organisms (e.g., Loire et al. 2013; Kutschera et al. 2020), and it is plausible that island populations in general exhibit lower *N̂*_*e*_ when compared to mainland species (Leroy et al. 2021). Furthermore, while inbreeding can be common in islands when compared to mainland populations (Frankham 1997, 2008), we did not detect any evidence of highly isolated small populations (beyond the main Maria/Southern populations split) that are particularly susceptible to loss of genetic diversity or inbreeding under the current patterns of connectivity, and overall genetic diversity is represented across remaining populations.

### Informing the conservation of genetic diversity

We suggest that the priority management actions for the conservation of genetic diversity in the forty-spotted pardalote should focus on increasing effective population size and maintaining or enhancing connectivity among the southern populations. These actions should be complemented by ongoing genetic monitoring to assess the future need for direct genetic management actions, such as translocations to enhance genetic diversity if there is a decline in genetic diversity. We argue that implementing effective habitat management on Bruny Is. and the adjacent Tasmanian mainland should be a priority. The populations in these areas are vulnerable to genetic stochasticity due to their small local population size and the presence of known threats such as habitat loss driven by land-clearing and tree dieback. Loss of habitat quality or connectivity would reduce effective population size and accelerate the loss of genetic diversity in the species. Most of the remaining populations are in areas of disturbed, young forest where nest site availability is low, leading to increased competition (Edworthy 2016a). Furthermore, parasitism from fly larvae on nestlings has been detected as a new threat, severely reducing recruitment (at least on North Bruny Is.; Edworthy et al. 2019). In addition, although our results do not allow us to infer the demographic effects of migrants, the asymmetric migration we detected (i.e., one-way migration off Bruny Is.) suggests the mainland sites may be sink habitats (Pulliam 1988; Gaggiotti 1996). Forty-spotted pardalotes were rediscovered in Southport in 2015 after having not been recorded at this site for >120 years (Webb et al. 2019). Given they were recently rediscovered, our results either indicate a recent recolonisation event from South Bruny and Partridge Is. or that Southport is functioning as a sink habitat given the low number of individuals detected at this site. Nonetheless, these dispersal events are encouraging and highlight the importance of Bruny Is. for maintaining adjacent populations, and the potential for habitat and threat management to lead to population expansion via colonisation. For Southport, provided there is enough area and habitat restoration is undertaken to address limiting factors (e.g., provisioning of nest boxes, investigate for the presence of parasites), translocating individuals from South Bruny Is. and Partridge Is. might be an effective strategy if migration rates are too low. For Tinderbox, habitat restoration may be sufficient to maintain the population, as we detected a 24% immigration rate in Tinderbox.

### Considerations for using translocation as a conservation tool

Conservation translocation has been proposed for forty-spotted pardalotes to create insurance populations (Webb et al. 2019) and our genetic assessment can be used to inform translocations that consider genetic diversity (e.g., Weeks et al. 2015). Given that North Bruny Is. and Maria Is. hold most genetic diversity in the species, these populations could be used to source founders following the ‘genetic-capture’ approach for threatened species described by Weeks et al. (2011, 2015). Provided that other aspects that impact reintroductions are accounted for (e.g., variation in vocal dialect, mitigation of threats; Parker et al. 2012; Valderrama et al. 2013; Martins et al. 2018; Lewis et al. 2021), sourcing founders from both populations should be considered to increase genetic diversity (Weeks et al. 2011) and reduce the demographic impact on the source populations. This is an important consideration because intensive collection of founders can negatively impact the remaining source population for years after collection occurs (e.g., Morrison et al. 2020). Although mixing populations for genetic rescue effect has often been avoided due to fears of outbreeding depression (i.e., loss of fitness when genetically different populations are crossed), the risk is generally low and often predictable (Frankham et al. 2011, 2019). Moreover, outbreeding depression primarily occurs due to adaptive differentiation (Frankham et al. 2011), which is not the case in most threatened species where differentiation is likely to be driven by fragmentation followed by interrupted gene flow, resulting in a small effective population size and drift (Reed and Frankham 2003; Lowe et al. 2005; Coleman et al. 2013; Weeks et al. 2015). There is also growing empirical evidence that genetic rescue can increase genetic diversity and population viability (Frankham 2015; Whiteley et al. 2015; Kronenberger et al. 2017; Ralls et al. 2018; Fitzpatrick et al. 2020).

While our study is important for informing discussion about the feasibility and need for translocations, there remain multiple unresolved conservation problems and unknown behavioural factors (e.g., song difference among populations). Forty-spotted pardalotes are habitat specialists, so habitat assessment and management should be undertaken prior to any translocation because unoccupied habitat is not necessarily suitable habitat (Osborne and Seddon 2012). Failing to remove or manage known threats will lead to failure even if the best genetic strategy is used to inform translocation. More importantly, if there is insufficient funding, we argue that conservation efforts should focus on managing the southern populations to increase their effective population sizes by expanding the availability of suitable habitat, before attempting translocations. Likewise, even though we did not consider the Flinders Is. population in this study, our results suggest that this subpopulation is likely to constitute its own conservation management unit. However, little is known of the status of pardalotes on Flinders Island and confirming whether they (i) still survive and (ii) are viable, should be a high priority. If the Flinders Is. population is extant but genetically diminished, it may be a good candidate for habitat restoration and translocation from other populations.

## Conclusion

Our study has important implications for the management of forty-spotted pardalotes and shows that by assessing contemporary genetic aspects, valuable information for conservation planning and decision-making can be produced to guide management actions (Hohenlohe et al. 2021). For example, careful planning prior attempting translocation that has been proposed for pardalotes is crucial because the species does not have a captive-bred population; thus, harvesting will rely on wild populations that have a small effective population size as shown in this study and are already under environmental pressure due to existing threats. Incorporating genetic monitoring alongside other management actions will inform appropriate interventions and increase the chances of species recovery. The compounding effects of environmental, demographic and harmful genetic processes can create an extinction vortex (Gilpin and Soulé 1986), and failing to account for the genetic problems may undo other good conservation actions. The neglect of genetic factors in species management needs to change (Ralls et al. 2018) because most species are not driven to extinction before the impact of harmful genetic processes is manifested (Spielman et al. 2004). Moreover, climate change will bring new challenges to species management, and genetically diverse populations have a greater potential to adapt (e.g., Bitter et al. 2019). Information gained from genetic data enriches the knowledge of the demographic processes that shape small populations and provide managers with defensible information that they can use to optimise their conservation strategies.

## Supplementary information

Supplementary material

Interactive map

S1

## Data Availability

The data and R scripts supporting the results in the paper are archived in Dryad 10.5061/dryad.zw3r228cc.
