# Supplementary material for: Using conservation genetics to prioritise management options for an endangered songbird
Source: Heredity (Edinb). 2023 Apr 5;130(5):289–301. doi: 10.1038/s41437-023-00609-6 (PMC10162965; doi:10.1038/s41437-023-00609-6)
Supplement: Supplementary file 1 — Supplementary material [file 41437_2023_609_MOESM1_ESM.docx]

**Supplementary material**


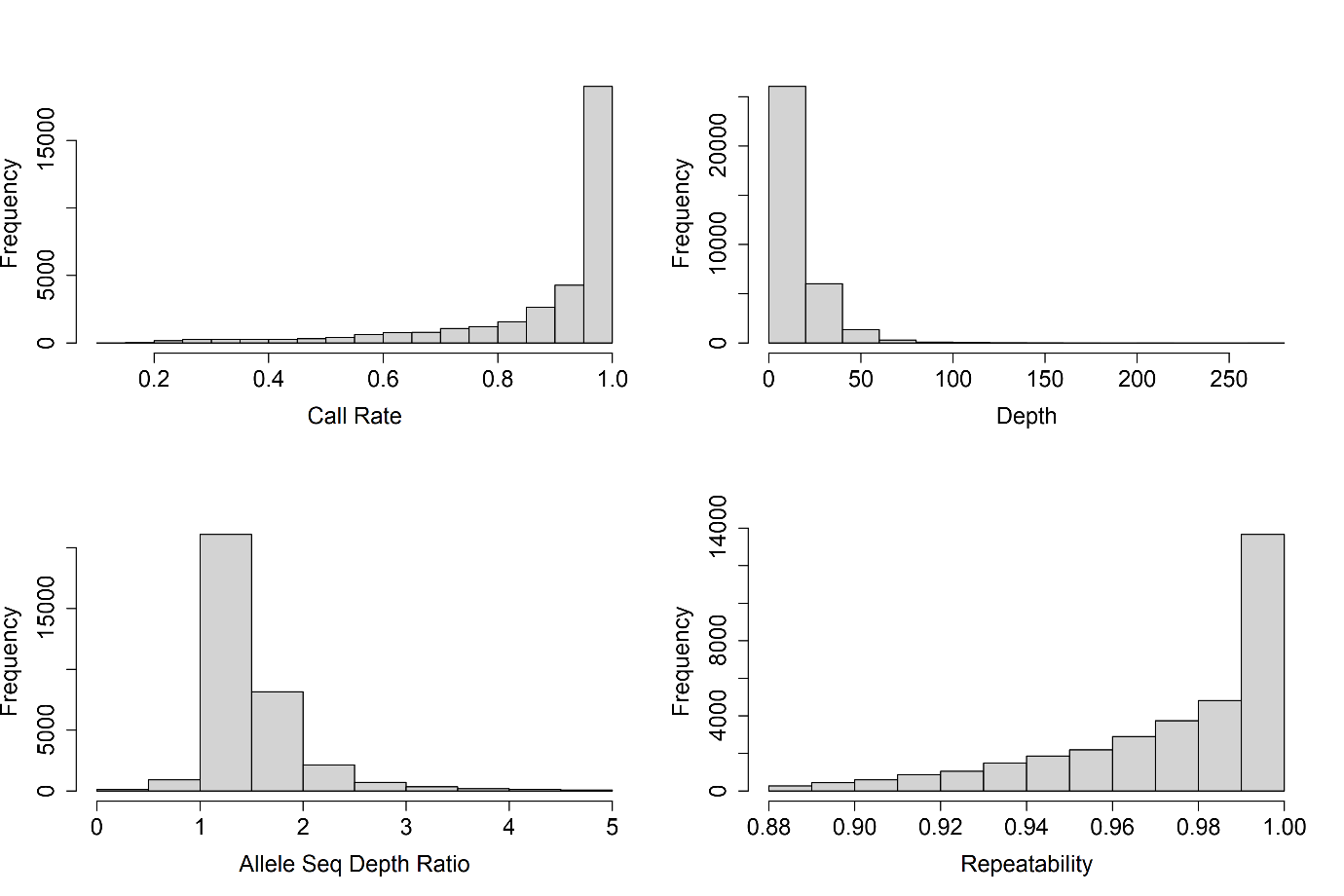


Figure S1. SNP metrics pre filtering


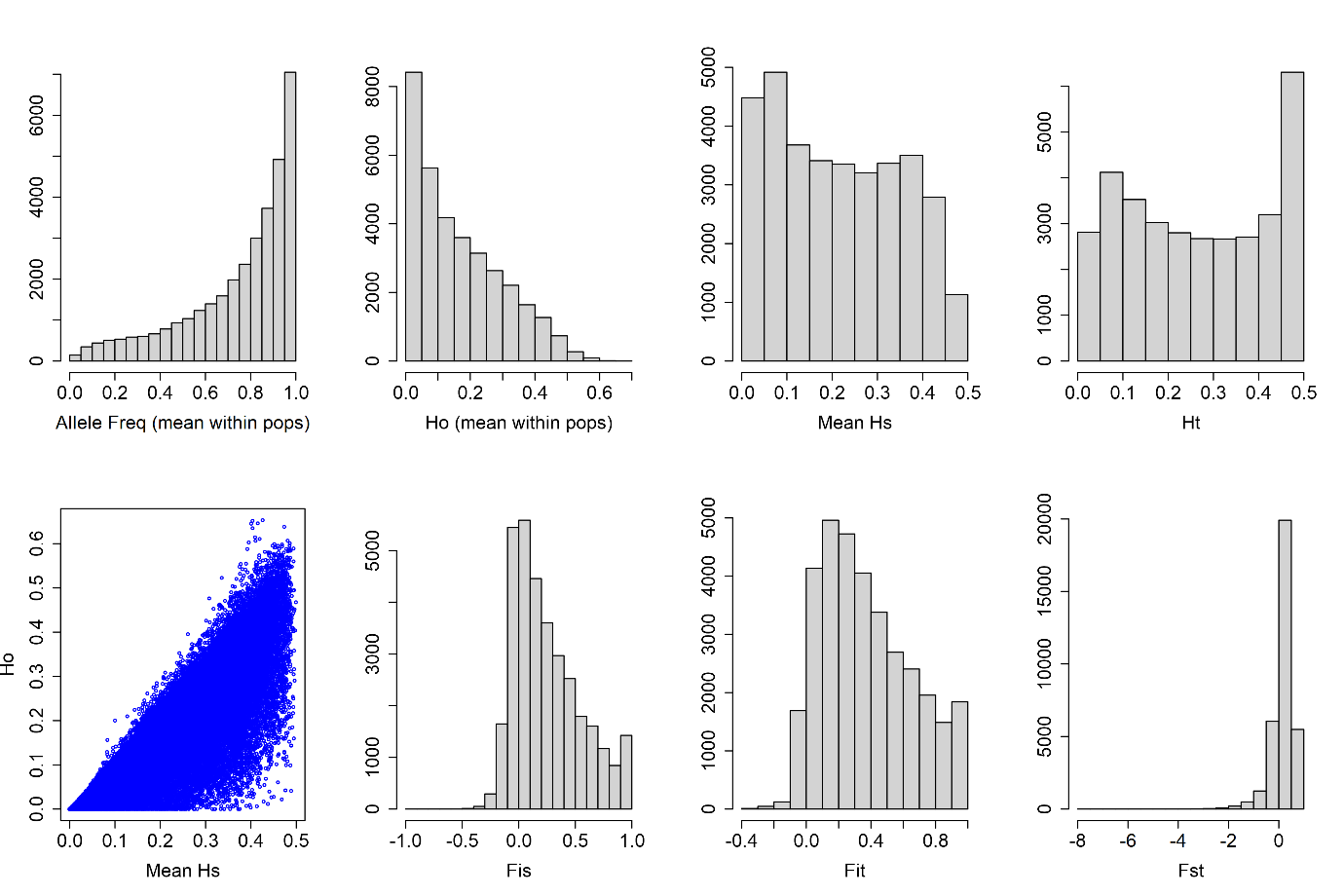


Figure S2. FST pre filtering


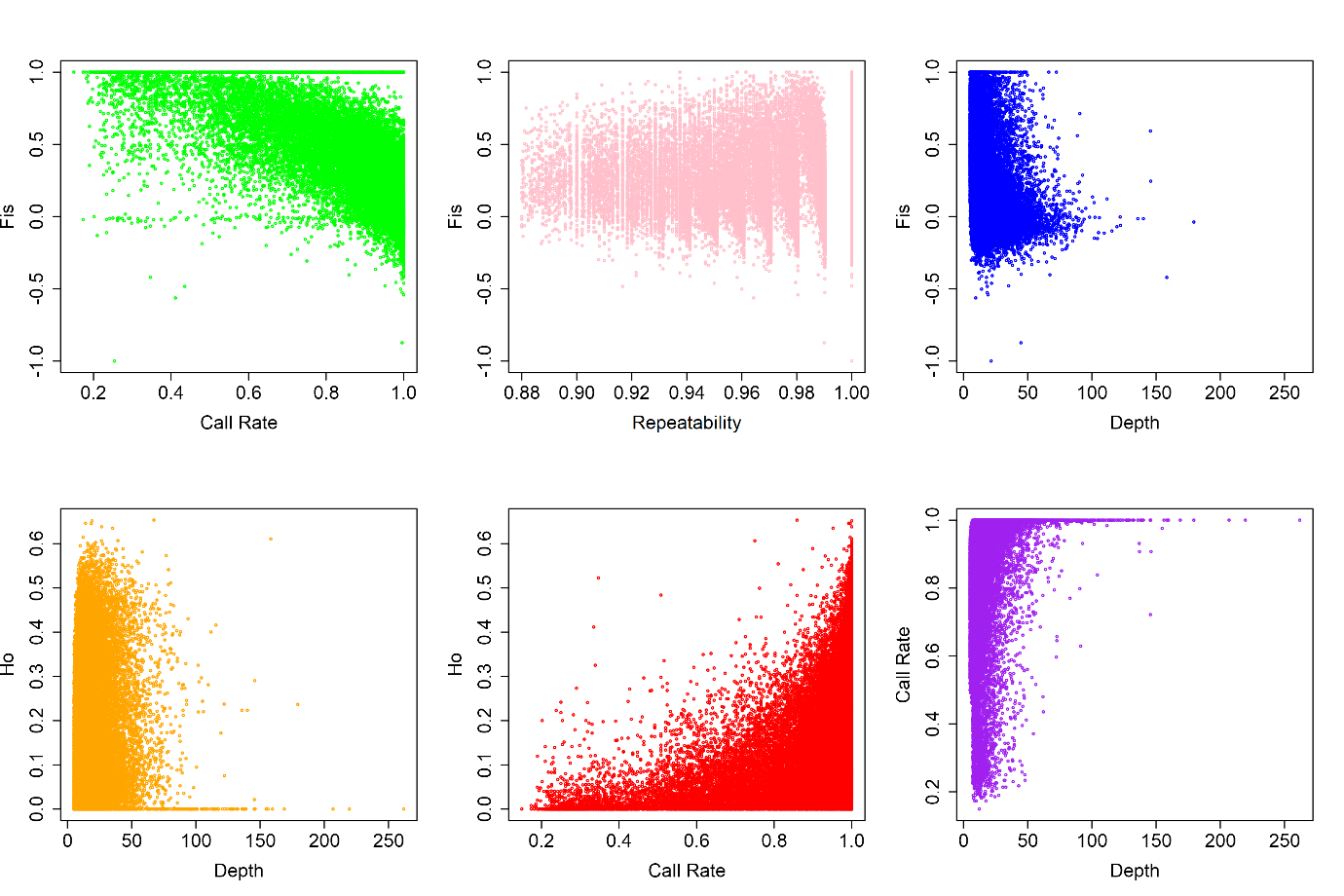


Figure S3. Population genetic diversity statistics against SNP quality pre filtering.


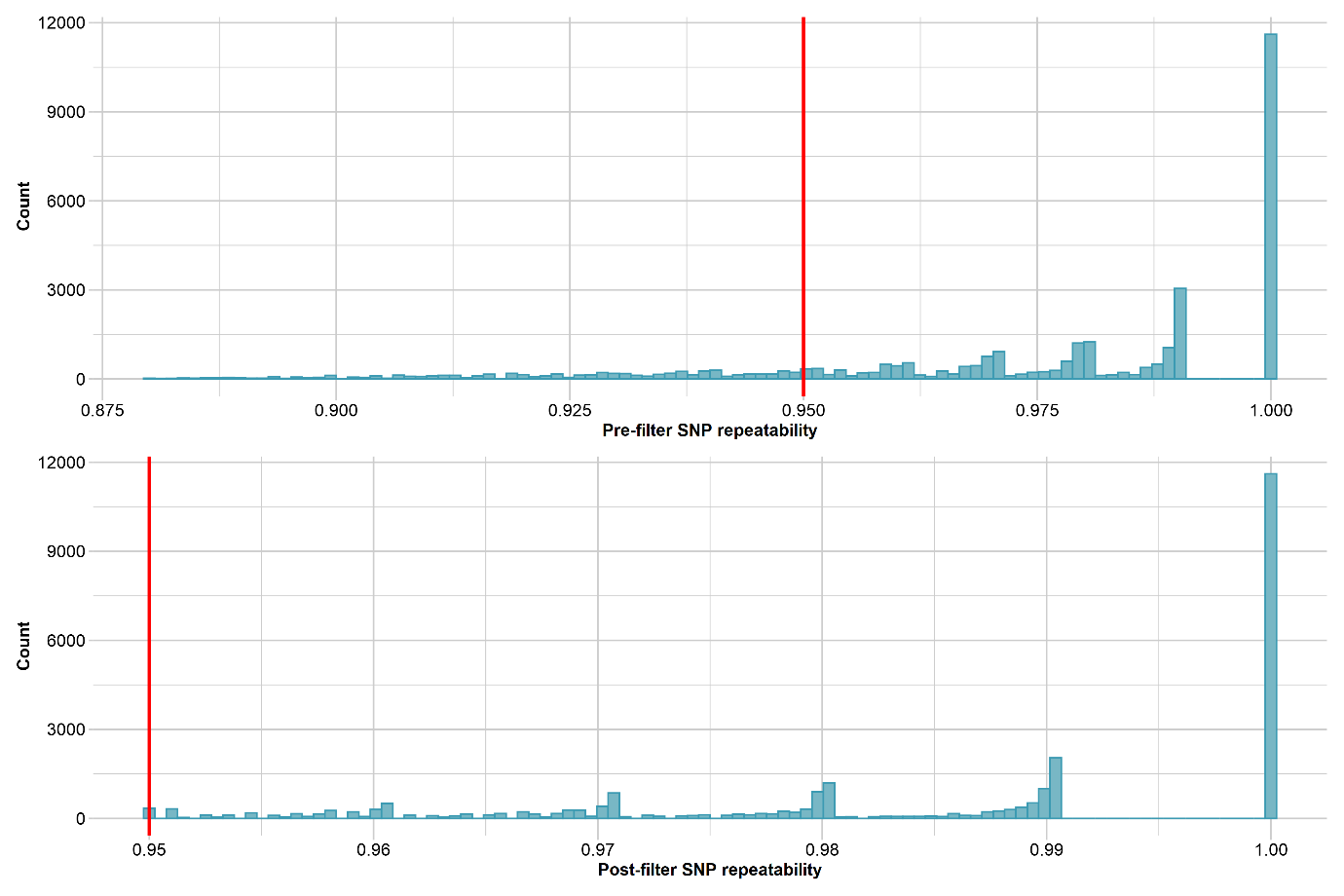


Figure S4. Pre and post filtering at 95% repeatability.


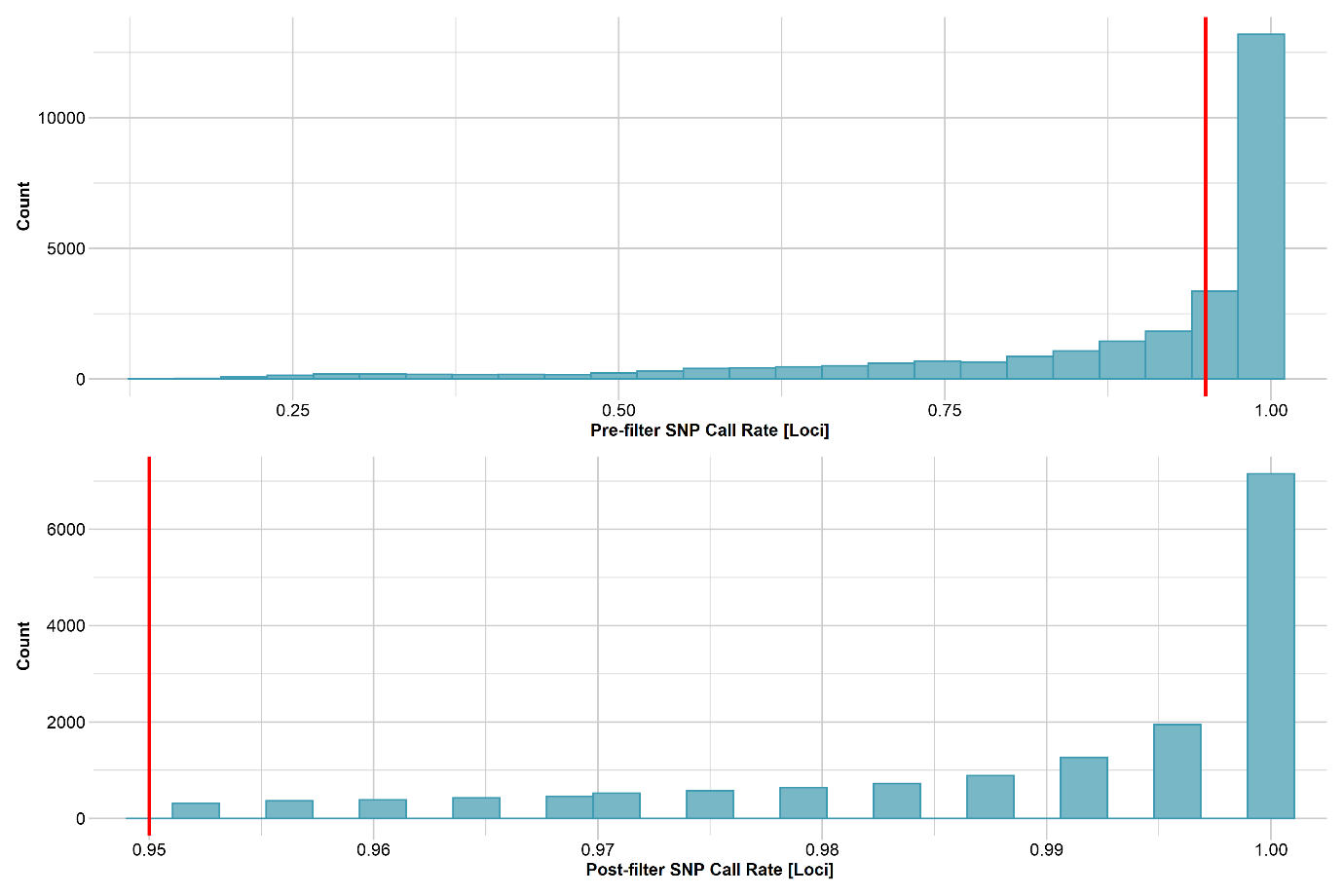


Figure S5. Pre and post filtering at 95% on loci call rate.


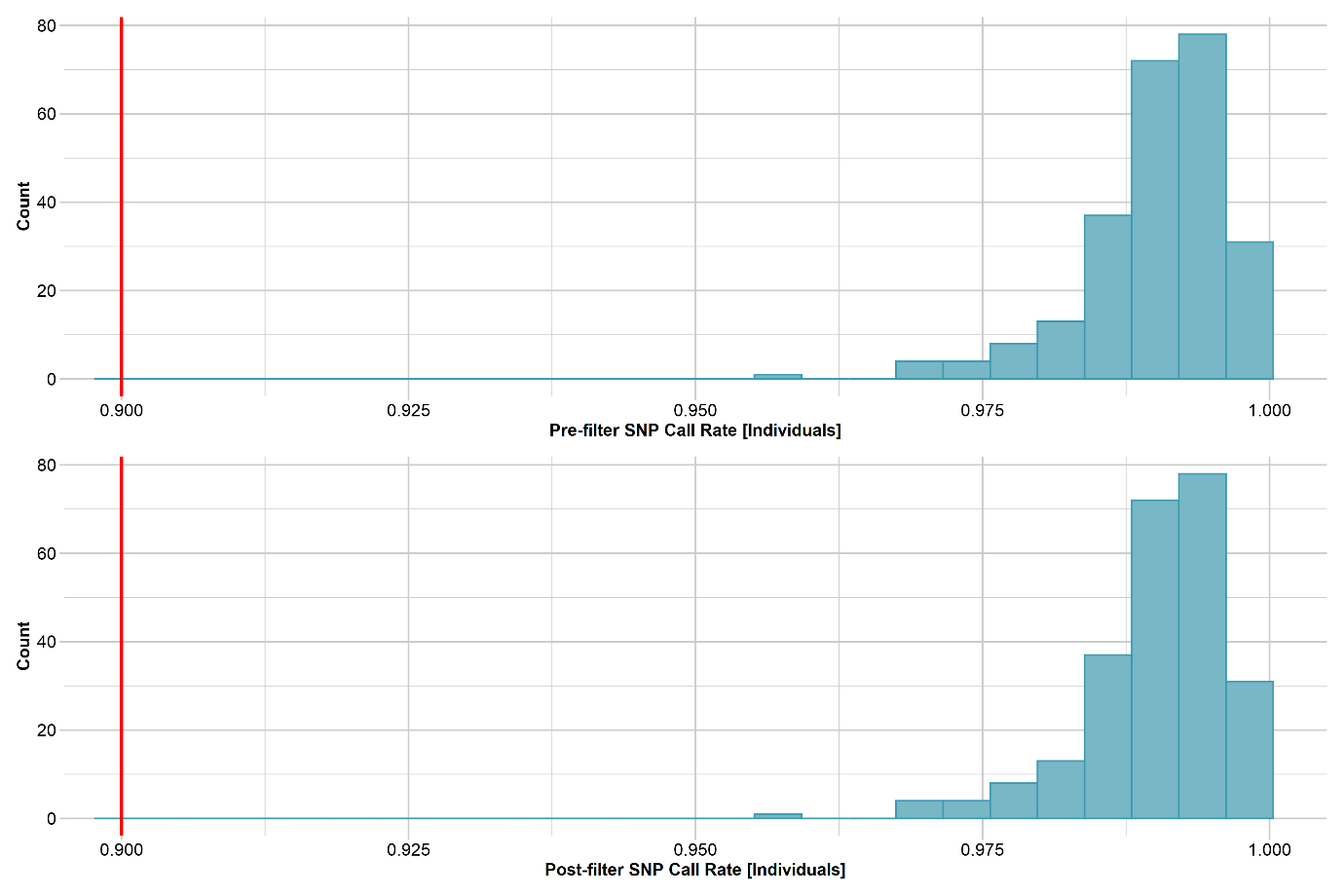


Figure S6. Pre and post filtering at 90% on individuals call rate.


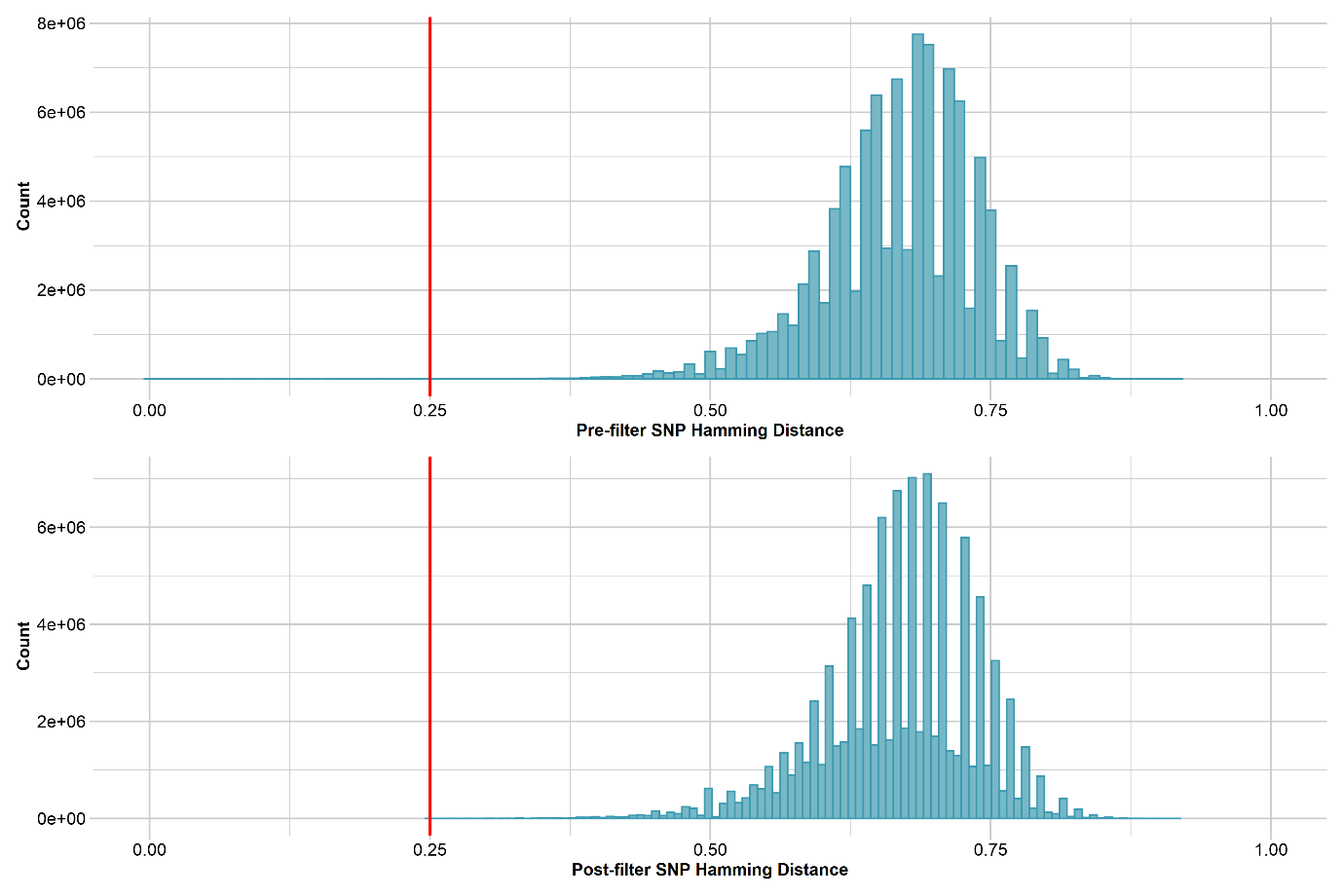


Figure S7. Pre and post filtering on pairwise Hamming distance.


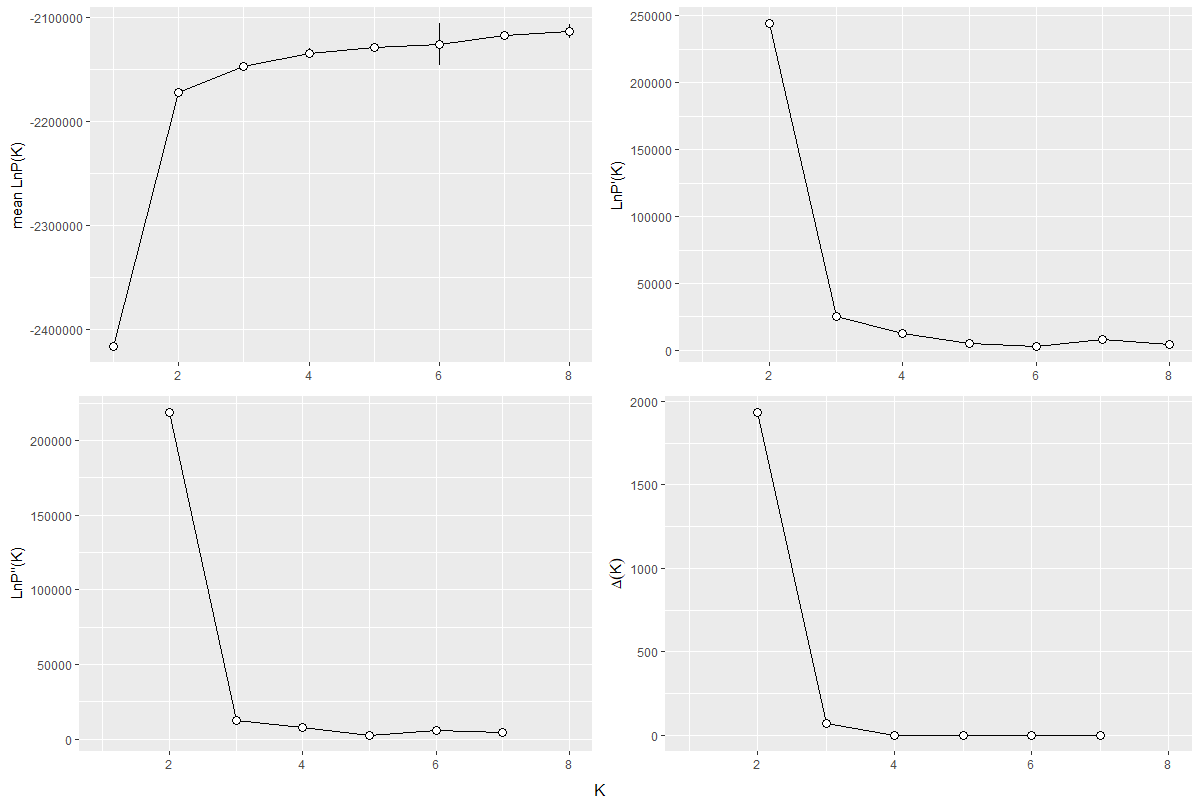


Figure S8. Diagnostic plots of the model with correlated allele frequency. First and second order changes in the likelihood for K (number of groups) for STRUCTURE analysis following Evanno (2005).
